# Blood Transfusion is an Independent Risk Factor for Postoperative Serious Infectious Complications After Pancreaticoduodenectomy

**DOI:** 10.1007/s00268-016-3553-7

**Published:** 2016-05-16

**Authors:** Liyang Zhang, Quan Liao, Taiping Zhang, Menghua Dai, Yupei Zhao

**Affiliations:** General Surgery Department, Peking Union Medical College Hospital, Chinese Academy of Medical Science, No. 1 Shuaifu Garden, Dongcheng District, 100730 Beijing, China

## Abstract

**Background/purpose:**

Blood transfusionhas been considered as a risk factor for postoperative infection after major surgery. However, the relationship between perioperative blood transfusion and the development of serious infections after pancreaticoduodenectomy remains controversial. The purpose of this study was to analyze risk factors associated with postoperative serious infections following pancreaticoduodenectomy.

**Methods:**

We conducted a retrospective study of 212 patients who underwent pancreaticoduodenectomy during past 2 years and assessed the risk factors for serious infectious complications.

**Results:**

Serious infections developed in 61 patients (29 %) including 47 cases of surgical site infection (SSI), 19 cases of bacteremia, and 13 cases of pneumonia. One patient died of severe septic shock. A multivariate logistic regression analysis of perioperative factors identified that pancreatic fistula (*P* < 0.01, OR = 9.763) and blood transfusion (*P* < 0.01, OR = 3.216) were significant risk factors for serious infections. After excluding 46 patients with pancreatic fistula, blood transfusion continued to be an independent risk factor for serious infections (*P* < 0.01, OR = 5.831).

**Conclusion:**

Blood transfusion was the strongest independent factor for serious infections after pancreaticoduodenectomy, which should be considered a quality indicator for the performance of pancreaticoduodenectomy.

## Introduction

Postoperative mortality rates after pancreaticoduodenectomy have substantially declined (<3 % in high volume centers) in the past decade, with improvements in surgical techniques and perioperative care [1]. However, the incidence of postoperative complications such as infectious complications continue to remain as high as approximately 50 % [2]. Serious infections such as surgical site infection (SSI), bacteremia, and pneumonia often prolong hospital stay increase medical expenses and occasionally cause mortality [3]. Furthermore, blood transfusion had been reported to be associated with infectious complications after major surgery [4–7]. However, the relationship between perioperative blood transfusion and the development of serious infections after pancreaticoduodenectomy remains undetermined. The purpose of this study was to evaluate risk factors for serious infections after pancreaticoduodenectomy.

## Patients and methods

Data of 212 patients who underwent pancreaticoduodenectomy in the General Surgery Department of Peking Union Medical College Hospital between January 2013 and December 2014 was collected. By chart review, perioperative data including age, gender, body mass index (BMI), nutritional screening scores (NRS-2002), clinical presentation, preoperative laboratory findings and biliary drainage procedures, type of resection, operative blood loss, red blood cell transfusion, and histopathological findings were recorded. For patients with obstructive jaundice, preoperative biliary drainage was performed by percutaneous transhepatic cholangiodrainage or endobiliary stent placement through an endoscopic route. All patients received prophylactic intravenous antibiotics (Ertapenem, 1.0 g) 30 min before skin incision. This regimen was continued for 48–72 h postoperatively. A total of 212 patients underwent pancreaticoduodenectomy alone or combined with other organ resection. Reconstruction was conducted by a modified Child method, and the pancreaticojejunostomy was established by an end-to-side duct to mucosa anastomosis with an external stenting tube inserted into the pancreatic duct. Blood transfusion was indicated when Hb levels of patients was lower than 10 g/L or HCT was lower than 30 % during or 24 h after the operation. A pancreatic fistula was defined as drain amylase level was >3 times the upper limit of normal serum amylase level on postoperative day three, according to the ISGPF2005 criteria [8].

Postoperative serious infectious complications include (1) SSI including incisional and organ/apace SSI, (2) bacteremia, and (3) pneumonia. SSI was diagnosed according to the modified definitions of surgical wound infection provided by the Centers for Disease Control and Prevention (CDC) National Nosocomial Infections Surveillance (NNIS) system [9]. Bacteremia was defined as positive blood cultures with high-grade fever (over 38.5 °C). Pneumonia was defined as a radiographic lung injury associated with pulmonary infiltrate with positive sputum cultures. When a serious infection was suspected, cultures from the drainage fluid, wounds, blood, or sputum were collected.

All statistical analysis was performed using SPSS 13.0 software. Continuous variables were expressed as mean ± standard deviation, and means were compared between groups using Student’s *t* test. Univariate analysis was conducted using Pearson *X*^2^ test. A logistic regression model for multivariate analysis was used to determine independent risk factors. A *P* value ≤ 0.05 was considered statistically significant.

## Results

Among the 212 patients who underwent pancreaticoduodenectomy, 78 (37 %) patients developed postoperative infectious complications (Table 1). Among these 212 patients, 61 (29 %) patients developed serious infections including 47 (22 %) patients with SSI, 19 (9 %) patients with bacteremia, and 13 (6 %) patients with pneumonia. Furthermore, one (0.5 %) patient died of multiple organ failure due to aggressive septic shock.

**Table 1:** Patient characters and perioperative outcomes

Age	59 years (22–81)
Gender	
Male F	128 (60 %)
Female	84 (40 %)
BMI	23.1 kg/m^2^ (15.6–31.0)
NRS-2002	
<3	72 (34 %)
≥3	140 (66 %)
DM	57 (26.9 %)
Disease	
Malignant tumor	170 (80 %)
Benign tumor	42 (20 %)
Preoperative biliary drainage	75 (35 %)
Blood loss	711 ml (200–8000)
Pancreatic fistula	46 (22 %)
Serious infections	61 (29.0 %)
Intra-abdominal infection	45 (21.2 %)
Wound infection	7 (3.3 %)
Bacteremia	19 (9.0 %)
Pneumonia	13 (6.1 %)
Other infections	17 (8.0 %)
Cholangitis	16 (7.5 %)
Catheter infection	10 (4.7 %)
Mortality	1 (0.5 %)

Various perioperative factors were compared between patients with and without serious infection (Table 2). The difference in pancreatic fistula (*P* < 0.01) and blood transfusion (*P* < 0.01) between the two groups was statistically significant. Multivariate logistic regression analysis results indicated that pancreatic fistula (*P* < 0.01, OR = 9.763) and blood transfusion (*P* = 0.003, OR = 3.216) were independently correlated with serious infections (Table 3).

**Table 2:** Results of the univariate analysis of perioperative factors associated with serious infection

Variables	No serious infection (*n* = 151)	Serious infection (*n* = 61)	*P*
Age (years)	58.4 ± 11.5	59.2 ± 11.2	0.68
Gender			0.38
Male (*n* = 128)	94	34	
Female (*n* = 84)	57	27	
BMI	22.7 ± 3.4	23.7 ± 3.7	0.06
NRS-2002			0.23
<3 (*n* = 72)	55	17	
≥3 (*n* = 140)	96	44	
Diabetes mellitus			0.41
Yes (*n* = 57)	43	14	
No (*n* = 155)	108	47	
Hb (g/dl)	12.6 ± 1.9	12.1 ± 2.3	0.08
WBC (×10^3^/mm^3^)	7.11 ± 2.8	7.89 ± 3.2	0.08
Lymphocytes (×10^3^/mm^3^)	1.35 ± 0.6	1.35 ± 0.7	0.99
Albumin (g/dl)	3.8 ± 0.6	3.6 ± 0.6	0.045
Total bilirubin (mg/dl)	3.9 ± 4.5	5.3 ± 6.0	0.10
BUN (g/dl)	14.4 ± 5.2	13.2 ± 4.7	0.28
Preoperative biliary drainage			0.09
No (*n* = 137)	103	34	
Percutaneous (*n* = 21)	11	10	
Endoscopic (*n* = 54)	37	17	
Pancreatic fistula			0.00
Yes (*n* = 46)	18	28	
No (*n* = 166)	133	33	
Blood loss (ml)	724 ± 680	681 ± 490	0.70
Red blood cell transfusion			0.003
Yes (*n* = 95)	58	37	
No (*n* = 117)	93	24	
Benign or malignant			0.12
Benign (*n* = 42)	34	8	
Malignant (*n* = 170)	117	53	
Extended resection			0.06
Yes (*n* = 29)	25	4	
No (*n* = 183)	126	57

**Table 3:** Results of the multivariate analysis of risk factors associated with serious infection

Variables	*B*	OR	95 % CI	*P*
Pancreatic fistula	2.279	9.763	4.091–23.297	0.000
Transfusion	1.168	3.216	1.478–7.000	0.003
Combined resection	−19.948	0	0	0.998
Constant	−2.106	0.122		

Patients without pancreatic fistula were divided into two groups according to the existence of serious infections. Among the 33 patients with serious infections, 23 patients had SSI, 15 patients had bacteremia, and 10 patients had pneumonia (Table 4). A multivariate analysis of pre- and intra-operative factors revealed that blood transfusion was significantly associated with the development of serious infections (*P* < 0.01, OR = 5.831; Table 5). The most frequently identified organisms in the study were Enterobacter species, followed by Enterococcus species.

**Table 4:** Results of the univariate analysis of pre- and intra-operative factors associated with serious infection

Variables	No serious infection (*n* = 133)	Serious infection (*n* = 33)	*P*
Age (years)	58.7 ± 11.4	59.2 ± 12.5	0.84
Gender			0.25
Male (*n* = 128)	83	17	
Female (*n* = 84)	50	16	
BMI	22.7 ± 3.2	23.9 ± 3.7	0.05
NRS-2002			0.48
<3 (*n* = 72)	49	10	
≥3 (*n* = 140)	84	23	
Diabetes mellitus			0.33
Yes (*n* = 57)	35	6	
No (*n* = 155)	98	27	
Hb (g/dl)	12.5 ± 1.9	12.6 ± 2.1	0.91
WBC (×10^3^/mm^3^)	7.12 ± 2.8	7.88 ± 3.4	0.24
Lymphocytes (×10^3^/mm^3^)	1.35 ± 0.6	1.44 ± 0.9	0.61
Albumin (g/dl)	3.8 ± 0.6	3.7 ± 0.6	0.57
Total bilirubin (mg/dl)	4.0 ± 5.3	5.5 ± 6.4	0.21
BUN (g/dl)	14.1 ± 6.5	13.8 ± 4.1	0.84
Preoperative biliary drainage			0.12
No (*n* = 137)	91	19	
Percutaneous (*n* = 21)	9	6	
Endoscopic (*n* = 54)	33	8	
Blood loss (ml)	761 ± 802	650 ± 307	0.49
Red blood cell transfusion			0.00
Yes (*n* = 95)	53	25	
No (*n* = 117)	80	8	
Benign or malignant			0.31
Benign (*n* = 42)	31	5	
Malignant (*n* = 170)	102	28	
Combined resection			0.12
Yes (*n* = 29)	20	1	
No (*n* = 183)	113	32

**Table 5:** Results of the multivariate analysis of pre- and intra-operative risk factors associated with serious infection

Variables	*B*	OR	95 % CI	*P*
Transfusion	1.763	5.831	2.377–14.309	0.000
Combined resection	−2.279	0.102	0.013–0.835	0.033
Constant	−3.036	0.048		

## Discussions

Although pancreaticoduodenectomy has a century of history and has been commonly performed in large medical centers, it remains as one of the complex abdominal surgeries with high morbidity rates [10–12]. Furthermore, the development of postoperative infectious complications remains a significant problem. The present study revealed that 37 % of patients had infectious complications, which is similar to previous reports. The exact risk factors for postoperative infection after pancreaticoduodenectomy remain controversial. In addition, multiple factors such as BMI (>25), poor nutrition, and operative time have been reported to be related to postoperative infections in few studies; among these factors, pancreatic fistula was reported as the most common independent risk factor associated with the high incidence of infectious complications [13]. Furthermore, our study revealed that the incidence of serious infection in patients with pancreatic fistula (61 %) was 3× higher than that in patients without pancreatic fistula (20 %). Moreover, for patients without pancreatic fistula, we found that 20 % of patients still developed serious infections. A multivariate analysis of perioperative factors revealed that blood transfusion was significantly associated with the development of serious infections, except for pancreatic fistula (*P* < 0.01, OR = 5.831). For pancreatic fistula, despite the advances in surgical techniques, it continues to haunt pancreatic surgeons, because none of the methods recommended for preventing pancreatic fistula have been conclusively proven to be effective. The reason for this is because its occurrence depends on several factors, and pancreatic texture might be an important factor [14] that could not be changed. Therefore, this was not discussed in detail in this study.

Blood transfusion has been recognized as a significant risk factor for minor and major complications in non-pancreatic surgeries. Janssen et al. [15] reported that the odds ratio for exposure to allogeneic blood transfusion in patients undergoing lumbar spine surgery was 2.6 for any postoperative infection. Xiao H et al. [16] also found that perioperative blood transfusion was an independent risk factor (OR = 2.71) for postoperative infectious complications after radical gastrectomy in patients with gastric cancer. However, controversy remains on the impact of blood transfusion on the outcomes of patients with pancreatic cancer. Ball et al. previously reported that transfusion of red blood cells after pancreaticoduodenectomy is linearly associated with 30-day morbidity [17]. However, Sutton et al. found that blood transfusion was not associated with the increased rate of infectious complications [18]. Therefore, we conducted this retrospective clinical study to investigate risk factors related to serious infections in an attempt to reduce postoperative infectious complications. Multiple factors including age, gender, body mass index (BMI), nutritional screening scores (NRS-2002), clinical presentation, preoperative laboratory findings and biliary drainage procedures, type of resection, operative blood loss, red blood cell transfusion, and histopathological findings were analyzed in the 212 patients who underwent pancreaticoduodenectomy and revealed that blood transfusion was found to be an independent risk factor associated with postoperative infections. The results of this study indicated that patients who received blood transfusion had a 3.2 times higher risk of developing serious infections than those without. Our study results also show that although patients with serious infections almost had the same volume of blood loss as those without, a larger percentage of patients (61.7 vs. 38.4 %) received blood transfusion in the serious infection group than in the non-infection group. Furthermore, considering the volume of blood transfused, 21.6 % of patients with serious infections had blood transfusions of more than 2 units, while only 11.5 % of patients without serious infections had more than 2 units of blood transfusion.

Except for transfusion errors (i.e., receiving the wrong blood) and transfusion-transmissible infections, increased risk of infections after blood transfusion is thought to be a result of transfusion-related immunosuppression [7, 19, 20]. Transfusions increase suppressor T cell activity and inhibit natural killer cell activity [21, 22]. Furthermore, the mitogenic activity of platelet-derived growth factors increases during storage of blood and may stimulate tumor growth following transfusion [23]. Therefore, perioperative transfusion may stimulate tumor growth directly or by an immunosuppressive effect, thereby having an adverse effect on patient survival [24].

Although blood transfusion is necessary in complex pancreatic procedures for severe blood loss, it has been reported that a substantial portion of perioperative transfusions among patients with PD did not meet predetermined criteria [25]. Therefore, strict criteria should be defined based on assessing the appropriateness of blood transfusion and limiting perioperative transfusions should be recommended. Choi reported pancreaticoduodenectomy could be performed successfully without blood transfusion in selected patients [26]. Our results also show that only less than 50 % of patients in this study had perioperative transfusion. Therefore, blood transfusions in pancreatic surgery might be reduced by minimizing blood loss, preoperative blood donation, and reinfusion.

This study has limitations. First, it is a retrospective study and selection bias could not be avoided. Second, although there were clear guidelines on the criteria of transfusion in our hospital, it may be influenced by individual surgeon or anesthesiologist preference. Finally, postoperative infections may be associated with the volume of blood transfused, which was not taken into consideration in the present study.

In conclusion, our study suggests that blood transfusion was the strongest independent factor for serious infections after pancreaticoduodenectomy, which should be considered a quality indicator for the performance of pancreaticoduodenectomy.
